# Decreased Connectivity in Precuneus of the Ventral Attentional Network in First-Episode, Treatment-Naïve Patients With Major Depressive Disorder: A Network Homogeneity and Independent Component Analysis

**DOI:** 10.3389/fpsyt.2022.925253

**Published:** 2022-05-27

**Authors:** Liqiong Luo, Xijun Lei, Canmin Zhu, Jun Wu, Hongwei Ren, Jing Zhan, Yongzhang Qin

**Affiliations:** ^1^Department of Oncology, Tianyou Hospital Affiliated to Wuhan University of Science and Technology, Wuhan, China; ^2^Department of Neurology, The First People's Hospital of Jiangxia District, Wuhan, China; ^3^Department of Neurosurgery, Wuhan Central Hospital Affiliated to Tongji Medical College, Huazhong University of Science and Technology, Wuhan, China; ^4^Department of Medical Imaging, Tianyou Hospital Affiliated to Wuhan University of Science and Technology, Wuhan, China; ^5^Department of Endocrinology, First Affiliated Hospital of Gannan Medical University, Ganzhou, China

**Keywords:** major depressive disorder, ventral attentional network, rest-state fMRI, network homogeneity, attentional network test

## Abstract

**Background and Objective:**

The ventral attentional network (VAN) can provide quantitative information on cognitive problems in patients with major depressive disorder (MDD). Nevertheless, little is known about network homogeneity (NH) changes in the VAN of these patients. The aim of this study was to examine the NH values in the VAN by independent component analysis (ICA) and compare the NH values between MDD patients and the normal controls (NCs).

**Methods:**

Attentional network test and resting-state functional magnetic resonance imaging (rs-fMRI) data were collected from 73 patients, and 70 NCs matched by gender, age, and education years. ICA and NH were employed to evaluate the data. Moreover, the NH values were compared, and Spearman's rank correlation analysis was used to assess the correlations with the executive control reaction time (ECRT).

**Results:**

Our results showed that the first-episode, treatment-naive MDD patients had decreased NH in the right precuneus (PCu) and abnormal ECRT compared with NCs. However, no significant correlation was found between the NH values and measured clinical variables.

**Conclusion:**

Our results highlight the potential importance of VAN in the pathophysiology of cognitive problems in MDD, thus offering new directions for future research on MDD.

## Introduction

Major depressive disorder (MDD) is a heterogeneous psychiatric disorder and one of the leading causes of disability and morbidity worldwide. MDD causes various symptoms such as persistent depression, loss of interest, a pervasive loss of pleasure, reduced energy, and cognitive function disturbance. A central cognitive impairment in MDD patients is the inability to allocate attention to appropriate emotional cues (1, 2). Compared with non-depression patients, depressed adult patients pay more attention to negative stimuli (3). In addition, although depressed adults tend to process sad stimuli similarly as non-depressed individuals, they have a habit of focusing on them a few seconds longer (1). The same attention bias can also be observed in adolescent depression (4). Neuropsychological studies have shown that attention function had been also impaired in patients with remitting MDD (5, 6). However, the neurobiological mechanism underlying the symptoms of MDD has not been fully understood.

Recent evidence has indicated that MDD may affect neural networks that are critical for the brain's development (7, 8). It has even been suggested that MDD itself is a brain network disease. For example, the default mode network, attention network, etc (8, 9). The ventral attention network (VAN) is associated with the orientation toward new, and unexpected stimuli. Its key brain regions are the inferior parietal lobule, anterior insula, temporal-parietal junction, inferior frontal gyrus, and middle frontal gyrus (10). By applying resting-state functional magnetic resonance imaging (rs-fMRI), studies have shown that depression was associated with abnormal VAN function. Furthermore, a meta-analysis of resting-state functional connectivity of MDD suggested that MDD was associated with hypoconnectivity between VAN and precuneus, and extended to occipital lobe and posterior cingulate cortex (11). Another rs-fMRI meta-analysis showed that the temporal, parietal junction activation was lower in patients with depression but higher in the ventro-lateral prefrontal cortex (VLPFC) than in healthy controls (12). In addition, many studies have shown that antidepressants and course of disease have a certain effect on the brain function and structure of MDD patients (13, 14). Therefore, investigating VAN's neurobiology in the early stage of disease and untreatment may be crucial for understanding the etiology of depression. However, the specific damage to the functions of brain regions within the VAN remains poorly understood.

Resting-state fMRI (rs-fMRI) is a widely applied approach for examining the functional alterations in neuropsychiatric disorders (9, 15). It is an advanced and non-invasive neuroimaging technique, which can quickly generate functional maps of the whole brain in the absence of task-related processing. Rs-fMRI has been extensively used in basic and clinical neuroscience research (16–21). At present, rs-fMRI is often used to investigate spontaneous neural activities and functional connectivity networks in the pathogenesis of MDD. This kind of research has been focused on group-level differences between study and control populations.

Independent component analysis (ICA) is a widely used data-driven approach for processing fMRI data. It enables the optimized capture of each matrix factor to make them as independent as possible. It has been successfully applied to fMRI data analysis and other types of biomedical data (22). Data-driven investigation of brain networks has received great attention in fMRI studies. It can help capture the spontaneous fluctuations in the interesting structures representing brain activities. ICA can be applied to investigate the specific damage to brain regions' functions. On this basis, analysis of network homogeneity (NH) can be used to further explore the changes in the functions of different brain regions of the patients. NH, which estimates the homogeneity of brain networks, uses biased forum assessment of MDD. It has been acknowledged that as a mental disorder, depression involves multiple brain regions and systems (23, 24). This simple, easy, and unbiased method reveals brain networks related to clinical applications that are worthy of research.

To the best of our knowledge, no previous studies have used ICA-based approaches to explore NH value differences in VAN for MDD. In this study, we applied rs-fMRI to explore the NH value differences of the VAN in the human brain. We tested the following hypotheses: (1) whether rs-fMRI can be used to identify the VAN in MDD; does VAN has different NH values in the first-episode, treatment-naive patients with MDD; (2) whether VAN has different NH values in certain specific brain regions; (3) whether different NH values in MDD are associated with certain functional changes.

## Materials and Methods

### Subjects

The study involved 73 patients with first-episode, treatment-naive depression without comorbid anxiety disorders, and 70 control participants without brain injuries or neurological diseases. All participants were from the Department of Psychiatry and Neurology, Tianyou Hospital Affiliated to Wuhan University of Science and Technology. Table 1 displays the demographic and clinical characteristics of the samples. The patients' diagnosis was conducted according to the Diagnostic and Statistical Manual of Mental Disorders (the 4th edition, DSM-IV) independently by two trained clinical psychiatrists (25). The depression severity of the patients was evaluated following the 17-item Hamilton Rating Scale for Depression (HRSD-17). All patients had a total score >17 in the HRSD-17 at MRI evaluation. Patients' exclusion criteria were the following: family history of neurological disorders, severe physical illnesses, substance abuse, left-handedness, children and adolescent, pregnancy, abnormal cerebral structures by initial MRI scanning, and the presence of other psychiatric disorders such as personality disorders or schizophrenia. For control participants, the exclusion standards were the same as those for MDD patients. Finally, 73 subjects with MDD and 70 healthy controls (matched for gender, age, education years) were included in the study.

**Table 1:** Characteristics of the participants.

**Demographic data**	**Patients (*n* = 73)**	**NCs (*n* = 70)**	**T (orx^**2**^)**	***P*-value**
Gender (male/female)	73 (43/30)	70 (40/30)	0.12	0.29^a^
Age (years)	26.32 ± 6.88	29.41 ± 5.31	1.74	0.84^b^
Years of education (years)	9.52 ± 0.93	9.52 ± 0.93	−1.33	0.62^b^
HRSD (score)	25.49 ± 7.10			
ECRT (ms)	91.60 ± 54.85	67.81 ± 48.02	2.13	0.04^b^

a *The p-value for gender distribution was obtained by chi-square test*.

b *The p-value were obtained by two sample t-tests*.

*NCs, normal controls; HRSD, Hamilton Rating Scale for Depression; ECRT, executive control reaction time*.

All participants signed completely informed consent and/or assent before participation in this study. This experimental design was approved by the ethics committee of Tianyou Hospital Affiliated to Wuhan University of Science and Technology and in accordance with the Declaration of Helsinki.

### Behavioral Paradigm

The attentional network test (ANT) designed by Fan et al. was performed using the Eprime and E-Studio software (Psychological Software Tools, Pittsburgh, PA, USA) (26). Image preprocessing and analyses mentioned below were performed according to a previously described approach (8). The standard procedures for ANT included three separate steps: (1) a “+” sign was placed in the central testing screen, which was considered as the fixation point. (2) A stimulus signal in the form of a target → or a foil^*^ was generated above or below the central screen. Four scenarios were set for the foils: no foil, one foil in the central part (one above the central screen and another below it, and one either above or below the central screen). (3) Arrows could be found in the following scenarios: one single arrow and five arrows in either one direction or different directions. The subjects were required to perform correct and quick target orientation. Then, ECRT was calculated by subtracting RT's consistent arrow direction from the inconsistent arrow direction RT. A higher ECRT value represented a lower efficiency of the executive control network.

The rs-fMRI images were obtained by using an Achieva 3T-MRI scanner (Philips, Ingenia). The scan lasted for 10 min. Patients were asked to lie down and close their eyes; they remained awake all the time. A prototype quadrature birdcage head coil filled with foam was utilized to minimize their head movement. The following parameters were used for functional imaging: slice thickness (5 mm), pitch (1 mm), flip angle (90?), a field of view (240 × 240 mm), and the ratio of repetition time to echo time (TR/TE) (2,000/30 ms). Meanwhile, the important settings were used for the structural scan (T1-weighted): repetition time (TR) = 20 ms, echo time (TE) = 3.5 ms, slice thickness = 1 mm, spin-echo sequence, and field of view (FOV) (= 24 × cm).

### Data Preprocessing

The data processing assistant for resting-state fMRI (DPARSF) software (27) in Matlab was used to precondition of the rs-fMRI imaging data. With the omission of the first 10 time points, slice time and head motion were rectified to adjust the time series of the images in order to ensure that the brain is in the same position for every image. No more than 2 mm of maximal displacement in the x, y, or z-axis and 2^?^ of maximal rotation were allowed for all participants. The structure of each patient was recorded to its functional image and partitioned, and a template was created in order to normalize the structures of the patients after they were defined following the Montreal Neurological Institute (MNI) standard template, the standardization process of the spatial deformation of the modulation and the structure of the voxel size using 1 × 1 × 1 mm^3^. Moreover, the use of the structure of each patient to the function of the conversion matrix was also standardized to the MNI space. In the functional image normalization, white matter signal, head motion parameters, and cerebrospinal fluid signal were taken as the removal covariates (Nuisance regression). The voxel size of 3 × 3 × 3 mm^3^ was utilized to resample. Subsequently, an 8 mm full width at half-maximum Gaussian kernel was used to smoother the obtained images. The images were bandpass filtered (0.01–0.1 Hz), followed by linear detrending to reduce the effect of low-frequency drifts and physiologic high-frequency noise. Several spurious covariates, including a signal from a region centered in the white matter, a signal from a ventricular ROI, and 6 head motion parameters obtained by rigid body correction, were excluded. The global signal removal might inevitably bring artifacts into the data and affect the resting-state connectivity patterns; besides, the global signal's regression might significantly influence the results when studying clinical cases (28). Therefore, the global signal was preserved to some extent.

### Ventral Attentional Network Identification

ICA was performed using the Group ICA utility to extract VAN components in templates from the GIFT fMRI toolbox (http://mialab.mrn.org/software/# gica) (29). The ICA analysis consisted of three steps from the GIFT toolbox: separation of independent components, data reduction, and back rebuilding. First, the optimal number of independent components (ICs) was estimated by the minimum description length criteria, which was 73 for MDD patients and 70 NCs. The data obtained from each participant were decomposed into spatially separated ICs using an algorithm, resulting in 73 independent spatial maps for MDD patients and 26 NCs. At last, the corresponding components for each participant were calculated. For each component, a statistical map and a threshold were set with the voxel-wise one-sample *t-*test. According to the Gaussian random field (GRF) theory, *p* < 0.01 was defined to represent a significant statistical modification of multiple comparisons, and voxel significance and cluster significance were defined at values of *p* < 0.01. Finally, the masks created for the parts in the VAN by combination were used for NH analysis.

### Network Homogeneity Analysis

The NH analysis results were calculated by an in-house script in Matlab. The VAN masks presented the correlation coefficients between the provided voxel and all others. The average correlation coefficient was defined as the homogeneity of a given voxel. Then, the average correlation coefficients were transformed into z values by z-transformation, which could significantly enhance the normal distribution. The obtained values were used to generate the NH map, which was finally subjected to z-transformation for group comparison.

### Statistical Analysis

The demographic information (age, gender, education years) and image data of the subjects were computed. A two-sample *t*-test was used to compare the continuous variables, and a Chi-square test to compare the classified data with the IBM SPSS Statistics 22.0 software. In order to measure the discrepancy in the NH regional group, a two-sample *t-*test was used to assist the individual-level NH map to the group-level voxel *t-*test analysis. Then, in the VAN mask, a two-sample *t*-test was used to analyze the NH maps through voxel-wise cross-subject statistics. GRF theory was employed to correct the significance levels for multiple comparisons. (GRF corrected, voxel significance: *P* < 0.001; cluster significance: *P* < 0.01).

NH values were extracted from the abnormal values in brain regions. Using the IBM SPSS Statistics 22.0 software, Pearson correlations analysis was performed after evaluating the data normality. A *p* < 0.05 was considered as statistically significant.

## Results

### Demographics and Clinical Features of the Subjects

The demographic information of the participants is shown in Table 1. No significant differences were found between the two groups in terms of gender, age, and years of education. Yet, the patient group had higher values of ECRT.

### VAN Maps Ascertained by Group ICA

**Figure 1:**
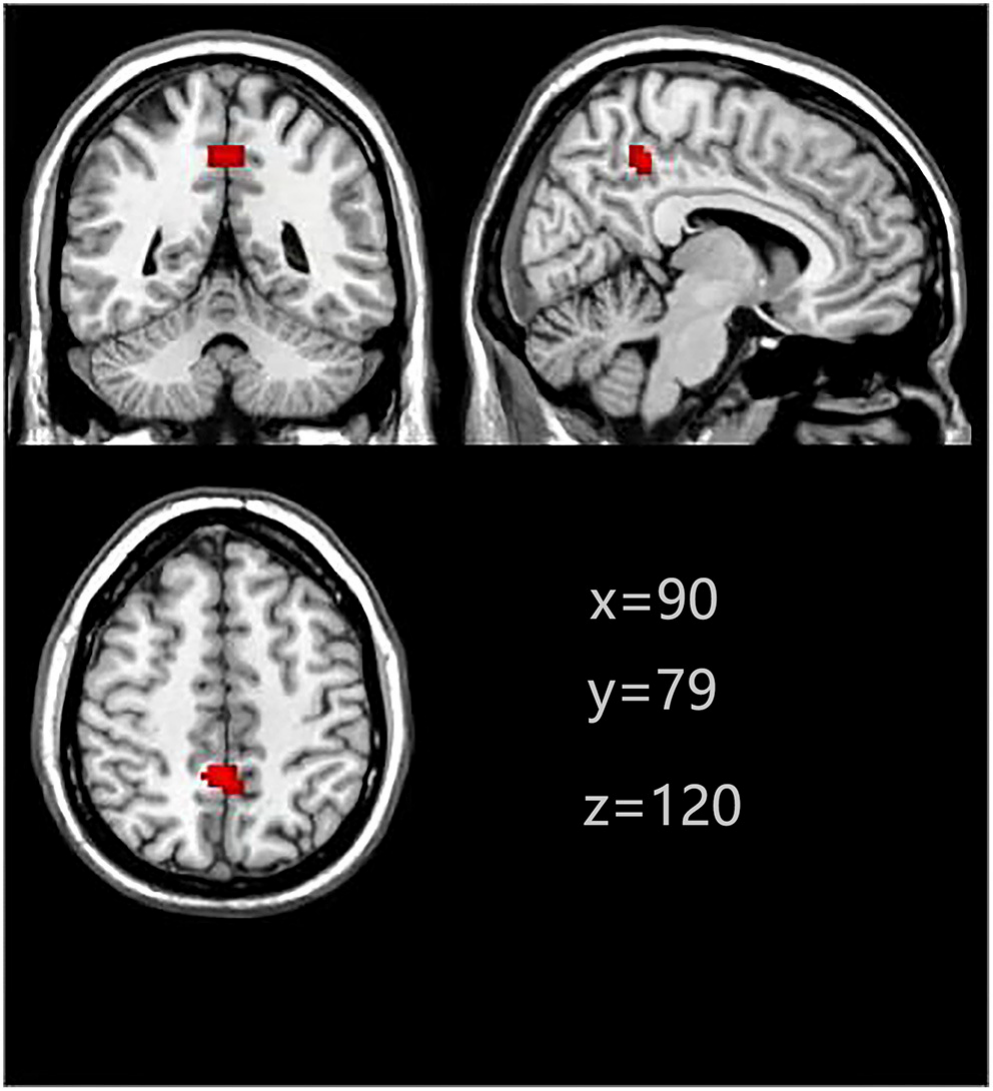
Ventral attentional network (based on group-ICA with a threshold at z≥5).

Two masks from MDD patients and NCs, respectively. Finally, the two masks were combined to generate a VAN mask. The parts involved in the VAN included the right PCu, right supper frontal gyrus, right inferior frontal gyrus, right supramarginal gyrus, left middle temporal gyrus, left inferior parietal gyrus, and left Cerebelum (Figure 1).

### Group Differences in VAN Regarding NH

**Figure 2:**
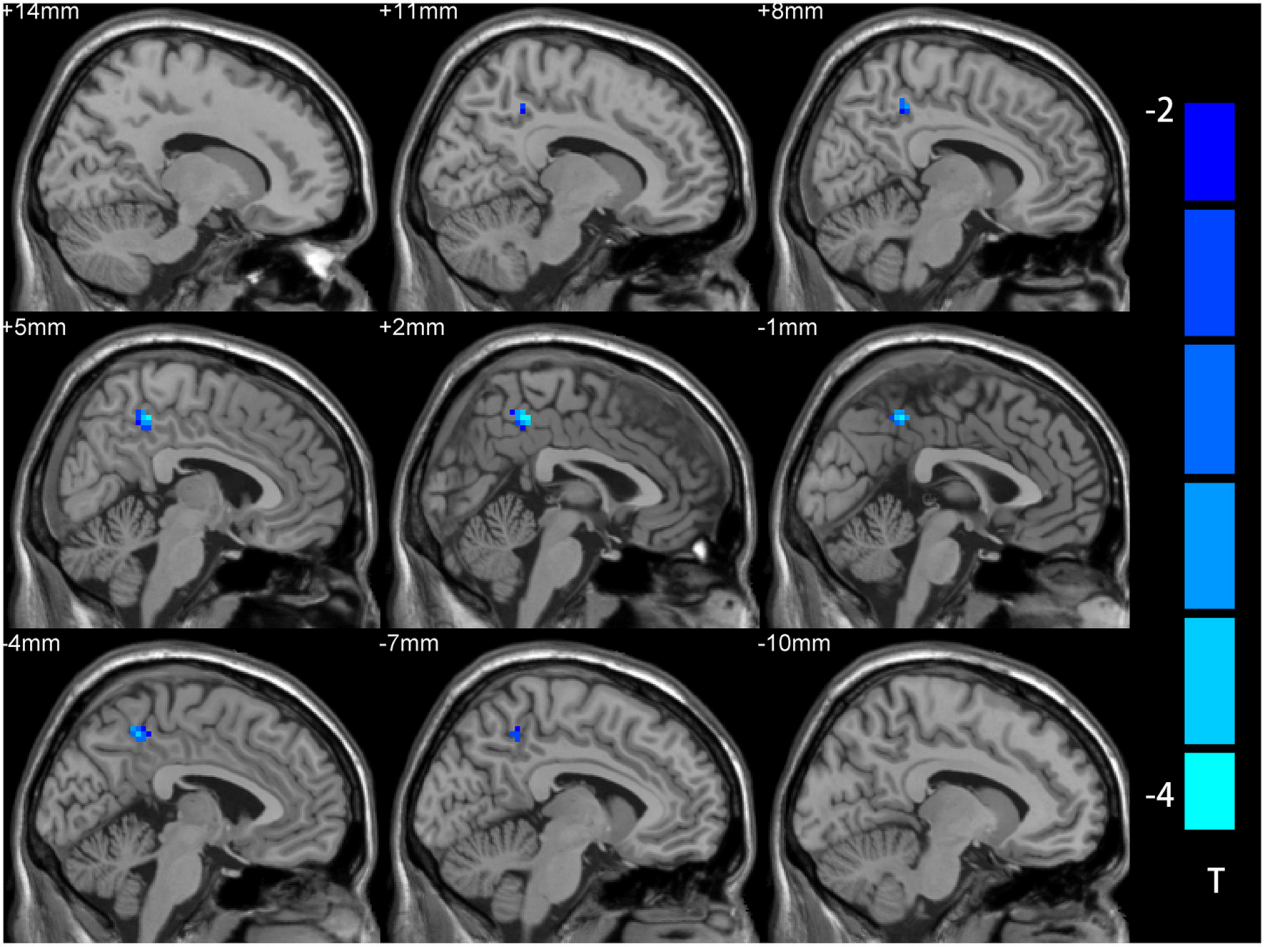
NH differences between the MDD patient and NCs in statistical maps. NH is lower in the right precuneus (PCu) of the MDD patients. Blue represents lower NH; color bars correspond to the T values obtained from the two-sample *t-*test. NH, network homogeneity; MDD, major depressive disorder; NC, normal controls; PCu, precuneus.

The two-sample *t-*test showed significant differences in NH values between the patient and control group within the VAN masks. Compared with NCs, the MDD patients had lower NH values in right PCu (Table 2 and Figure 2).

**Table 2:** Signification differences in NH values between the groups.

**Cluster location**	**Peak X**	**(MNI) Y**	**Z**	**Number of voxels**	**T value**
Patients < controls Right Pcu	3	−45	48	48	−3.57

*NH, network homogeneity; MNI, Montreal Neurological Institute; PCu, Precuneus*.

### Correlation of NH With Clinical Variables

Significant group discrepancies were found in the right PCu, from which the NH values were obtained. In the patient group, Pearson linear correlation analysis was performed to investigate the correlations between NH, ECRT, and illness severity. The results showed no significant correlation between NH and those clinical variables.

## Discussion

A number of studies have found that under the condition of selective attention, the neural function of normal aging will change (30, 31). Furthermore, many articles have reported alteration in the cerebral hemisphere's functional network due to other factors (32). However, little is known about the changes related to MDD in the ventral attention network (VAN), which is the basis of selective attention. This study examined MDD-related changes within the VAN, focusing on abnormality between its regions. We examined 73 patients and 70 control participants based on NH values of network signals from rs-fMRI as well as ERCT of their test performance. We identified the VAN independently for both groups using spatial independent component analysis. Three main findings emerged: first, rs-fMRI could be used to identify the VAN, and MDD patients had lower NH values than control participants. Second, MDD patients had similar connectivity among posterior regions compared to control participants but lower NH values among the right precuneus (PCu). Finally, MDD patients had a shorter ECRT compared to controls. Thus, this study suggests that neuro-functional changes in MDD affect VAN values. Our results revealed that anterior regions were of greater importance for MDD patients; especially the right PCu had enhanced centrality.

Currently, rs-fMRI research on MDD patients has identified a broad range of brain network abnormalities, but the underlying mechanism remains unclear (33, 34). Some studies have attributed the pathophysiological mechanism of depression to abnormal DMN connectivity (35, 36). However, these studies have only focused on the changes in DMN and have ignored the changes in other brain regions. VAN is an important mediator of stimulus-driven attention and a task-positive network that is activated during attentional orientation or reorientation. Multiple cortical areas, such as the middle and inferior frontal gyri, inferior parietal lobule, anterior insula, and temporal-parietal junction, have participate in this process (35, 37). Some previous studies have demonstrated that VAN is a key brain network for stimulus-driven attention. Still, it remains unclear how depression is related to effective connectivity within the VAN.

Recent rs-fMRI studies have shown that the PCu, orbitofrontal gyrus, and amygdala are the main cortical or brain junction areas. The PCu is an area of the brain involved in various complex cognitive functions, including self-processing, operations spatial imagery, and episodic memory retrieval. In MDD, spatial affect learning disorder is related to the PCu activation (38). Dysregulation as a neural substrate of depression, the PCu is involved in the formation of self-consciousness, which may be related to the low self-esteem of depression (39–41). The PCu is also associated with autobiographical memory dysfunction, which is another significant feature of MDD (42). The results suggest that PCu has a vital role in functional connectivity in mediating cognitive and affective functions. Herein, we utilized the rs-fMRI method and ICA analysis to explore the NH value of the PCu in the brain VAN of the patients with first-episode, treatment-naive depression. The right PCu showed statistically significant differences in NH between the MDD patients and the controls. To be more specific, the patients with first-episode, treatment-naive depression showed decreased NH values compared with the control participants. Previous studies have also confirmed the importance of VAN in the pathophysiology of psychiatric disorders (24, 43). Characterization of the network connectivity in the brain can help to further elucidate the specific functions of the PCu and shed new light on how PCu dysfunctions contribute to the clinical manifestation of MDD.

Reaction time (RT) is associated with increased amplitude response in cognitive control regions. Some research has suggested that RT-related activity can represent the amount of time taken to perform a decision process, which is relatively brief on fast RT trials and relatively long on slow RT trials. Executive control reaction time (ECRT) is a specific RT form, mainly focusing on executive control, which still needs to be evaluated in more detail. Here, we hypothesized that MDD patients have abnormal VAN homogeneity related to certain clinical variables such as ERCT. Therefore, based on previous work, we further explored whether there was a close correlation between a distinct decrease in the NH of VAN and abnormal ERCT. As a result, no significant correlation was found between NH and ERCT. In previous studies on the brain networks, there were either decreases or increases in the NH values in mental disorder patients (42, 44–46). However, in this study, the NH values exclusively showed decreases. This discrepancy may be attributed to the subjects in the present study who were first-episode, treatment-naïve depression patients without the establishment of a compensatory mechanism due to the absence of treatment.

Two neural systems for goal-directed and stimulus-driven attention, the dorsal attention network (DAN) and the VAN, have been described in the human brain. There are asymmetries in functional connection patterns related to these key nodes of the attention network. This asymmetric development pattern of the attention network may be a neural feature, i.e., with the development of attention mechanism, from the bottom-up over-representation to the greater top-down attention ability (47). Little is known about the relative uniqueness of these attention networks in the brain. Another brain system, known as the saliency network, has also been linked to functions that overlap with VAN functions, including responding to behavior-related stimuli. At the same time, some researchers suggested that the functional and anatomical overlap of the VAN and salience networks make part of the same system (48). Though there is an abundance of literature on human brain networks, little research focused on these networks' typical development and special function. This study selected the normal control participants and first-episode, treatment-naive MDD patients as the research subjects. We explored whether VAN has different NH values that were potentially more predominant in the first-episode, treatment-naive MMD patients.

This has a few limitations. First, this study was only focused on variations in the VAN and might have ignored some significant changes in other brain regions. The DAN, VAN, and the salence network are important intracranial neural networks, which cannot be completely separated from each other in organizational structure but are relatively independent or complementary to each other in function. Second, the current study was the deletion of the age factor, largely due to the unknown effects of puberty on developing the brain's attention networks. The third limitation was the influence of the recurrent factors, largely due to the unknown effects of the recurrent attacks on the brain's attention networks' cross-development. Forth, this study has a small sample size. However, our results elucidate the importance of VAN in the pathophysiology of MDD by exploring the abnormal NH values in the VAN of MDD patients.

At present, cognitive-behavioral therapy can improve network-level abnormalities with better treatment effects. At the same time, existing descriptions of the VAN lack detail and offer limited insight into the underlying structural connections of the network. This study explores the possible regulation model of PCu in the VAN and establishes a more effective treatment mode for MDD.

## Data Availability Statement

The original contributions presented in the study are included in the article/supplementary material, further inquiries can be directed to the corresponding authors.

## Ethics Statement

The studies involving human participants were reviewed and approved by the Ethics Committee of Tianyou Hospital Affiliated to Wuhan University of Science and Technology. The patients/participants provided their written informed consent to participate in this study.

## Author Contributions

LL conceived the structure of the manuscript and wrote the manuscript. XL, JW, HR, and JZ collected and analyzed the data. CZ and YQ conceived and critically reviewed the manuscript. All authors have read and approved the final manuscript.

## Funding

This research was supported by Health Commission of Hubei Province Scientific Research Project (Grant No. WJ2021M007), General Project of Science and Technology Department of Hubei Province (Grant No. 2020CFB512), the Science and Technology Plan of Health Commission of Jiangxi Province (Grant No. 202130648), and the Science and Technology Research Project of Department of Education of Jiangxi Province (Grant No. GJJ201522).

## Conflict of Interest

The authors declare that the research was conducted in the absence of any commercial or financial relationships that could be construed as a potential conflict of interest.

## Publisher's Note

All claims expressed in this article are solely those of the authors and do not necessarily represent those of their affiliated organizations, or those of the publisher, the editors and the reviewers. Any product that may be evaluated in this article, or claim that may be made by its manufacturer, is not guaranteed or endorsed by the publisher.
